# Supplementary material for: The impact of COVID-19 on cancer screening and treatment in older adults: The Multiethnic Cohort Study
Source: eLife. 2023 Jun 27;12:e86562. doi: 10.7554/eLife.86562 (PMC10642961; doi:10.7554/eLife.86562)
Supplement: Supplementary file 1. [file elife-86562-supp1.docx]

**Supplementary Table 1. Comparison of Ethnicity and Educational Level between 2019 MEC Cohort Survivors and 2021 COVID Survey Participants**

| **Ethnicity** | **(%)** | | **Educational Level**  **(%)** | | | | | | | |
| --- | --- | --- | --- | --- | --- | --- | --- | --- | --- | --- |
|  | **2019 MEC**  **Cohort**  **(N = 111,433)** | **COVID Survey Cohort**  **(N = 6,974)** | **≤8 years**  **_____________** | | **9–12 years**  **_____________** | | **Vocational**  **_____________** | | **At least some college**  **_____________** | |
| **Male and Female** |  |  | **MEC** | **COVID** | **MEC** | **COVID** | **MEC** | **COVID** | **MEC** | **COVID** |
| **White** | 23.1 | 43.8 | 1.7 | 0 | 18.8 | 6.8 | 4.3 | 2.4 | 75.2 | 90.8 |
| **Japanese American** | 28.2 | 32.3 | 1.3 | 0.1 | 27.4 | 7.0 | 13.8 | 9.0 | 57.5 | 83.9 |
| **Latino** | 23.6 | 7.6 | 35.3 | 8.1 | 30.9 | 20.4 | 6.8 | 5.9 | 27.0 | 65.6 |
| **Native Hawaiian** | 6.7 | 7.4 | 1.3 | 0.2 | 42.3 | 21.1 | 9.4 | 8.2 | 47.0 | 70.5 |
| **African-American** | 11.7 | 5.2 | 3.5 | 0 | 28.0 | 10.0 | 6.1 | 3.3 | 62.4 | 86.7 |
| **Other** | 6.7 | 3.7 | 10.0 | 0.4 | 22.3 | 10.6 | 6.9 | 4.7 | 60.8 | 84.3 |
