# Supplementary material for: The impact of COVID-19 on cancer screening and treatment in older adults: The Multiethnic Cohort Study
Source: eLife. 2023 Jun 27;12:e86562. doi: 10.7554/eLife.86562 (PMC10642961; doi:10.7554/eLife.86562)
Supplement: Supplementary file 2. [file elife-86562-supp2.docx]

**Supplementary Table 2. Distribution of Baseline COVID Survey Participants by Health Status, Comorbidities, and Medication Use, Hawai‘i and Los Angeles (N = 6,974)**

|  | **Male**  **N (%)** | **Female**  **N (%)** |
| --- | --- | --- |
| **Health Status:** |  |  |
| Has Health Problems that Require Them to Stay at Home | 104 (3.5) | 189 (4.9) |
| Missing | 35 | 64 |
|  |  |  |
| Needs Assistance with Daily Living Activities on a Regular Basis | 96 (3.2) | 156 (4.0) |
| Missing | 35 | 61 |
|  |  |  |
| Cane, Walker, or Wheelchair/Scooter Usage to Get About | 193 (6.4) | 363 (9.4) |
| Missing | 36 | 57 |
|  |  |  |
| History of Health Problems that Limit Activities | 591 (19.7) | 747 (19.3) |
| Missing | 36 | 62 |
|  |  |  |
| **Comorbidities** |  |  |
| History of Heart Disease | 622 (20.8) | 412 (10.6) |
| Missing | 38 | 69 |
|  |  |  |
| History of Hypertension | 1,441 (48.0) | 1,824 (47.1) |
| Missing | 37 | 63 |
|  |  |  |
| History of Diabetes | 548 (18.3) | 559 (14.4) |
| Missing | 35 | 63 |
|  |  |  |
| History of Lung Disease, COPD, or Asthma | 297 (9.9) | 523 (13.5) |
| Missing | 37 | 67 |
|  |  |  |
| History of Kidney Disease | 164 (5.5) | 175 (4.5) |
| Missing | 36 | 65 |
|  |  |  |
| Diagnosed with Cancer in the Past 5 Years | 478 (15.9) | 439 (11.3) |
| Missing | 35 | 62 |

**Supplementary Table 2. (Continued)**

| **Medication Use** |  |  |
| --- | --- | --- |
| Taking immunosuppressant medications (including steroids, methotrexate, biologic agents) | 110 (3.7) | 182 (4.7) |
| Missing | 44 | 78 |
|  |  |  |
| Taking heartburn medication Pepcid (famotidine) | 307 (10.3) | 383 (9.9) |
| Missing | 39 | 64 |
|  |  |  |
| Taking Aspirin (baby aspirin or std dose) | 1,135 (37.9) | 1,091 (28.1) |
| Missing | 36 | 59 |
|  |  |  |
| Taking NSAIDs like ibuprofen, nurofen, diclofenac, naproxen | 267 (8.9) | 418 (10.8) |
| Missing | 35 | 65 |
|  |  |  |
| Taking Blood Thinning Medication (such as Coumadin/Warfarin, Eliquis/apixaban) | 480 (16.0) | 293 (7.6) |
| Missing | 42 | 75 |
|  |  |  |
| Taking Blood Pressure Medication ending in -pril (such as enalapril, lisinopril, captopril, ramipril) | 771 (25.8) | 632 (16.4) |
| Missing | 46 | 84 |
|  |  |  |
| Taking Blood Pressure Medication ending in  –sartan (such as losartan valsartan, irbesartan) | 874 (29.3) | 1,004 (26.0) |
| Missing | 46 | 83 |
