# Supplementary material for: The impact of COVID-19 on cancer screening and treatment in older adults: The Multiethnic Cohort Study
Source: eLife. 2023 Jun 27;12:e86562. doi: 10.7554/eLife.86562 (PMC10642961; doi:10.7554/eLife.86562)
Supplement: Supplementary file 3. [file elife-86562-supp3.docx]

**Supplementary Table 3. Distribution for Postponing Regular Health Care Visits Due to COVID-19 Pandemic by Sex (N = 6,974)**

|  | **Male**  **N (%)** | **Female**  **N (%)** |
| --- | --- | --- |
| Other*: | 768 | 1416 |
| Dermatology | 156 | 218 |
| Eye | 248 | 411 |
| ENT | 44 | 67 |
| OB-GYN | 0 | 105 |
| Orthopedist | 36 | 62 |
| Podiatrist | 31 | 39 |
| Urologist | 70 | 27 |
| Neurologist | 27 | 28 |
| Rheumatologist | 15 | 50 |
| Pulmonologist | 19 | 35 |
| Audiologist | 24 | 50 |
| Allergist | 2 | 15 |
| Gastroenterologist | 5 | 10 |
| Laboratory Testing/Imaging | 21 | 140 |
| Physical/Massage/Chiropractic Therapy | 34 | 89 |
| Other specialists | 15 | 23 |
| Other healthcare providers | 21 | 47 |
|  |  |  |

*All other categories were sorted from the text responses for specifying the “Other specialist or health care provider.”
